# Sixteenth Annual Report of the State Commissioner in Lunacy for the Year 1888

**Published:** 1889-08

**Authors:** 


**Sixteenth Annual Report** of the State Commissioner in Lunacy for the year 1888. Transmitted to the Legislature January 15, 1889. Octavo, pp. 155. Albany: The Troy Press Company, printers. 1889. (Through Hon. Leroy Andrus.)

This is a very complete report of the question, involving the care of the insane in this State, as well as the present condition of their management. The report has been compiled under difficulties, as very little help could be had from the outgoing commissioner, and the recommendation that hereafter the Commissioner report to the Governor on or before the first day of December of each year, is timely. Since, however, the creation of the State Lunacy Commission, an annual report will, of course, be made. This very complet ereport will be of assistance to the new commission in organizing this work.

